# An Assessment of Pediatric Dental Caries and Family Quality of Life in an Informal Amazonian Community

**DOI:** 10.5334/aogh.3331

**Published:** 2021-08-20

**Authors:** Marc Horton, Sahar Zolfaghari, Eduardo Bernabé, Leann Andrews, Jorge Alarcón, Mauro Echevarría, Joseph Zunt, Ana Lucia Seminario

**Affiliations:** 1 University of Washington, US; 2 King’s College London, GB; 3 Universidad Nacional de la Amazonía Peruana, PE; 4 Universidad Peruana Cayetano Heredia, PE

## Abstract

**Background:**

Oral diseases are among the most prevalent non-communicable diseases worldwide, disproportionally affecting vulnerable populations. The Community of Claverito is one of many informal urban floating communities located on the Amazon River in Peru.

**Objectives:**

To assess child and caregiver dental health status (DHS) and to measure the associations between child DHS and child and family quality of life in the informal Community of Claverito.

**Methods:**

DHS, as measured by decayed and filled teeth (DFT/dft), was recorded for 66 children and 35 caregivers using the WHO Oral Assessment form. Oral health-related quality of life was measured using the Parental-Caregiver Perceptions Questionnaire (P-CPQ). The family impact of child oral disorders was measured using the Family Impact Scale (FIS). Descriptive statistics, correlations, and regression analyses were used to evaluate the associations between DFT/dft, P-CPQ, and FIS scores (p < 0.05).

**Findings:**

The majority of children assessed were female (52%) with a mean age of 9.4 years (SD ± 4.4). The prevalence of untreated child dental caries was 97%. The child and caregiver’s mean DFT/dft scores were 6.8 (SD ± 4.5) and 8.7 (SD ± 13.3), respectively. Mean total P-CPQ and total FIS scores were 33.4 and 12.5, respectively. A significant positive association was observed between child DFT/dft scores and total FIS scores (p < 0.01). Significant associations were also observed between child DFT/dft scores and caregiver age (p < 0.01) and child DFT/dft scores and caregiver DFT scores (p < 0.01).

**Conclusions:**

Children and their caregivers living in the Community of Claverito exhibited high levels of dental caries. Children’s untreated dental caries were associated with both family’s quality of life and caregivers’ untreated dental caries. Further research is needed on how improving availability and access to oral health services have the potential to benefit the health of residents of informal communities like the one of Claverito.

## Introduction

According to estimates from the 2015 and 2017 Global Burden of Disease Studies, oral disorders (including caries, periodontal disease, and edentulism) were the most prevalent diseases among 354 assessed conditions, and dental caries of the primary teeth affected more than 570 million children [1,2]. Oral disorders manifest as pain, impairment, and loss of function, and can affect individuals throughout their lifetimes. And, like other chronic diseases, oral diseases disproportionally affect the poor and socially disadvantaged. Inversely associated trends between oral health disparities and socioeconomic status are consistent in highly developed [3,4], developing [5], and underdeveloped countries [6], demonstrating that individuals of lower socioeconomic status experience higher levels of oral disease. According to the UN Human Settlements Programme (UN-Habitat), as of 2014, one in eight people globally lived in slums and lacked access to safe water, durable housing, sufficient living space, and security of tenure that prevents forced evictions [7]. Slum-dwelling residents often lack or are denied access to basic services, including medical and dental care [8]. Despite the rapid growth of these informal communities, and the social determinants of health predisposing these populations to health risks, these populations receive relatively limited attention in medical and oral health research [9].

Iquitos, with a population of approximately 422,000, is the largest city in the Peruvian Amazonian rainforest [10]. In recent years, the city has experienced a rapid increase of immigrants moving from the surrounding jungle seeking jobs, education, and healthcare. In the already dense city, migrants are forced to find or construct housing, which often results in the development of informal communities. Floating structures are built on the Amazon River floodplains, and these have become home for many immigrants. Due to such precarious housing and poor environmental conditions, residents of these communities experience chronic illnesses, vector-borne and infectious diseases, exposure to severe storms and flooding, and lack of water, sanitary infrastructure, and access to food [11]. In 2017, a collaborative group from the University of Washington (School of Dentistry, Department of Civil and Environmental Engineering, Department of Environmental and Occupational Health, Department of Global Health, Department of Landscape Architecture, Department of Neurology, and School of Nursing) partnered with the Centro de Investigaciones Tecnológicas Biomédicas y Medioambientales, the Universidad Nacional de la Amazonía Peruana, and the Instituto Nacional de Salud in Peru and developed a transdisciplinary action research program, InterACTION Labs, to gather baseline health data and design and implement built environment improvement projects aimed at advancing the health, community strength, and future development of residents of one of these informal river communities—the Community of Claverito [12,13]. As a contributor to InterACTION Labs, dental faculty collected information about the community’s oral health status and the impact of oral health status on the residents’ quality of life.

Few studies specifically report on the oral health status of slum-dwelling children and its impact on their oral-health related quality of life (OHRQoL) or their families’ oral-health related quality of life [14,15]. In order to develop sustainable and scalable population health interventions, community-level baseline assessments of both disease and quality of life are necessary. The purpose of this study was twofold: 1) to describe the prevalence and distribution of dental caries among the pediatric and caregiver populations of the Community of Claverito; and 2) to evaluate the relationships between the children’s dental health status and the OHRQoL of the children and the impact of the child’s condition on the family. Given the established association between socioeconomic status and the prevalence and severity of oral diseases [16], we hypothesized that the pediatric and caregiver population of the Community of Claverito would have a high level of untreated caries, and that the children’s dental health status would be associated with lower quality of life for both the children and their families.

## Methods

This study was approved by Ethics Committees at both the University of Washington (# STUDY00000022) and The Instituto de Medicina Tropical “Daniel Alcides Carrión” at the Universidad Nacional Mayor de San Marcos in Peru (#CIEI-2018-004). Informed consents were signed by the parents and caregivers of the pediatric participants, and participants ages 10–18 also provided informed assent, in accordance with the Declaration of Helsinki.

This was a population-based cross-sectional study of all children aged 0–18 years and their parents and caregivers from the Community of Claverito in Iquitos, Peru. The InterACTION Labs program gathered demographic data for all residents who lived at least 6 months of the year in the community. In 2018, there were 270 documented individuals living in the Community of Claverito, and 138 (51.1%) were children aged 0–18 years. The community was comprised of 50 houses, 44 of which had members of the household 18 years or younger. There were on average 2.7 children per household. All members of the community were invited to participate in this transdisciplinary study. Additional data collected on physical and mental health, nutrition, demographics, microbiome, environmental changes and other human and ecological health factors is being analyzed for further publications. For this manuscript, we are presenting data of community members 0–18 years of age and their caregivers.

The InterACTION Labs established a long-term partnership with Claverito, and projects were identified in response to a series of community participatory workshops in which residents identified their needs and priorities. Data was collected to support the analysis of these community-driven projects. Data collection was performed over two days in February 2018 in the participants’ houses. Informed consent/assent was obtained, and demographic information was collected on the pediatric participants and their caregivers. If a member of the family was not present on the first day of data collection, the team returned to reattempt data collection on the second day. Dentition status was recorded for participants 18 years and younger and for their caregivers using the WHO Oral Assessment form [17] by a calibrated team of public health dentists. When more than one caregiver was present, each family decided which adult would interact with the research team. The dental team was composed of six dentists and six dental students. Dentists (University of Washington and Universidad Nacional de la Amazonia Peruana) conducted the oral exams, and dental students (from local institution) recorded the findings. All team members participated in a half-day session at the Universidad Nacional de la Amazonía Peruana Dental School to review the oral health assessment form (paper form), practice the examination process (with headlights, gauze, dental mirrors, and dental probes), and standardize diagnoses and data entry protocols. Dental exams were conducted on 66 children and 35 caregivers. When a dental emergency was identified, the individual was referred to the local hospital where he/she received free treatment. Caregivers were interviewed to assess quality of life using the Parental-Caregiver Perceptions Questionnaire (P-CPQ) and Family Impact Scale (FIS), previously validated in Spanish [18,19]. One P-CPQ/FIS survey was administered per household.

The Peruvian Spanish validated P-CPQ [20] consists of two questions which measure global ratings of the child’s oral health and the impact of the child’s oral/orofacial condition on his or her overall well-being, and 31 questions representing four health domains: oral symptoms (six questions), functional limitations (eight questions), emotional well-being (seven questions), and social well-being (ten questions) (***Table 1***). Higher global ratings scores denote a poorer rating of the child’s oral health and a greater impact of the child’s oral health status on their overall well-being. The responses to the health domains questions are reported as separate domain scores and as a total score. The total score is the sum of the thirty-one health domains questions and can range from 0 to 124. Higher domain scores and total score indicate a greater degree of the impact of the child’s oral health status on their quality of life. The FIS [21] consists of 14 questions, which measure the impact of the child’s oral/orofacial condition on his or her family’s quality of life. The 14 questions are divided into four sub-scales: parental/family activity (5 questions), parental emotions (4 questions), family conflict (4 questions), and financial burden (1 question). The responses are reported as subscale scores and a total score, which can range from 0 to 56. Higher scores indicate greater impact of a child’s oral condition on family quality of life (***Table 1***).

**Table 1:** Summary of the Parental-Caregiver Perceptions Questionnaire (P-CPQ) and Family Impact Scale (FIS).


	NUMBER OF QUESTIONS	RANGE

**Global rating**	**2**	**0–8**

*Q1: Rate the health of the child’s teeth, lips, jaw, and mouth: “Excellent” = 0, “Very good” = 1, “Good” = 2, “Fair” = 3, “Poor” = 4*		

*Q2: Indicate how much the child’s overall well-being is affected by the condition of his/her teeth, lips, jaw, or mouth: “Not at all” = 0, “Very little” = 1, “Some” = 2, “A lot” = 3, “Very much” = 4*		

**P-CPQ Subscales**	**31**	**0–124**

**P-CPQ Subscales**		

Oral symptoms	6	0–24

Functional limitations	8	0–32

Emotional well-being	7	0–28

Social well-being	10	0–40

*How often in the last three months has the child experienced symptoms or discomfort due to the condition of their teeth, lips, mouth, and jaws: “Never” = 0, “Once or twice” = 1, “Sometimes” = 2, “Often” = 3, “Every day or almost every day” = 4*		

**FIS Total scale**	**14**	**0–56**

**FIS Subscales**		

Parental/family activity	5	0–20

Parental emotions	4	0–16

Family conflict	4	0–16

Financial burden	1	0–4

*During the last three months, how often* … *(has there been disagreement or conflict in your family)*… *because of your child’s condition’: “Never” = 0, “Once or twice” = 1, “Sometimes” = 2, “Often” = 3, “Every day or almost every day” = 4*		


Dentition status and P-CPQ and FIS surveys were administered verbally by Spanish-speaking Peruvian dental team members and were recorded on paper forms. Each individual received an ID number for research purposes. Manual double data entry was performed to transfer the records into a de-identified REDCap (Research Electronic Data Capture) [22] database hosted at the University of Washington. REDCap is a secure web application for building and managing online surveys and databases. Access to the database was limited to authors of this manuscript.

Statistical analyses were performed using the statistical software R, version 3.6.1 [23]. Descriptive statistics were calculated for all participants. Pearson correlation coefficients were calculated to evaluate the relationships between dental health status (DFT/dft scores) and P-CPQ domain and total scores as well as FIS subscales and total scores. Unadjusted and adjusted linear regression models with generalized estimating equations (GEE) were fit to the data to evaluate the relationships between demographic variables (child’s gender, child’s age, caregiver’s age, caregiver’s dental health status) and OHRQoL variables (which caregiver completed the OHRQoL survey, total P-CPQ score, and total FIS score) with child’s dental health status. GEE was utilized to account for clustering of children within households. Hypothesis tests were performed using a 5% significance level.

## Results

Dentition status and demographics were recorded for 66 community members 18 years and younger (response rate: 48%) and for 35 of their caregivers (response rate: 80%) (***Table 2***). Of the participants 18 years and younger, thirty-two were male (48%), and the mean age was 9.4 years (Standard Deviation [SD] ± 4.4). Untreated decay was observed in 97% of all pediatric participants, and the mean DFT/dft score was 6.8 (SD ± 4.5). The mean age of the caregivers was 37.7 years (SD ± 13.3), and the mother was the primary caregiver for the majority of the children (83%). The caregiver’s mean DFT score was 8.7 (SD ± 13.3), and 27% of caregivers had a DFT score greater than 11.

**Table 2:** Demographics and dental health status of child and caregiver.


	TOTAL N N (%)

**All Children n**	66 (100%)

Sex	

Male	32 (48.5%)

Female	34 (51.5%)

Child Age categories	

0 to 5 years	14 (21.2%)

6 to 11 years	30 (45.5%)

12 to 18 years	22 (33.3%)

DFT/dft categories	

0 to 4 teeth	24 (36.4%)

5 to 9 teeth	24 (36.4%)

10 to 20 teeth	18 (27.3%)

Untreated dental decay*	64 (96.9%)

**All Caregivers**	35 (100%)

Caregiver age categories	

18 to 30 years	11 (31.4%)

31 to 40 years	12 (34.3%)

41 to 76 years	12 (34.3%)

Caregiver DFT categories	

0 to 6 teeth	12 (34.3%)

7 to 11 teeth	12 (34.3%)

> 11 teeth	9 (25.7%)

Missing data	2 (5.7%)

Surveys completed by	

Mother	29 (82.9%)

Other caregiver	6 (17.1%)

	**Mean** ± **SD; Median (IQR)**

Child age, yr, mean ± SD, median (IQR)	9.4 ± 4.4; 9.0 (6.0, 13.0)

Child DFT/dft, mean ± SD, median (IQR)	6.8 ± 4.5; 6.5 (3.0, 10.0)

Caregiver age, yr, mean ± SD, median (IQR)	37.7 ± 13.3; 35.0 (29.0, 42.0)

DFT, mean ± SD, median (IQR)	8.7 ± 4.7; 9.0 (5.0, 11.5)


DFT/dft: Decayed filled teeth. * Primary or permanent dentition.

One P-CPQ/FIS survey was administered per household, and data was recorded for 35 households. In 18 of the households, the P-CPQ/FIS survey was completed on behalf of two or more children. P-CPQ/FIS data were available for 64 children with DFT/dft data. The P-CPQ scores ranged from 1 to 84, with a mean score of 33.4 (SD ± 20.0) (***Table 3***). A positive correlation was observed between child DFT/dft scores and total P-CPQ. Positive correlations were also observed between DFT/dft scores and global rating of oral health, global rating of overall well-being, and the P-CPQ domains emotional well-being and social well-being. Negative correlations were observed with the P-CPQ domains oral symptoms and functional limitations. The associations between P-CPQ scores and child DFT/dft scores were not statistically significant.

**Table 3:** P-CPQ and FIS scores and correlation of scores with child DFT/dft.


	MEAN ± SD, MEDIAN (IQR)	CORRELATION

**Parental-Caregiver Perceptions Questionnaire**		

Global Rating of Oral Health (0–4)	3.0 ± 0.7, 3.0 (3.0, 3.0)	0.08

Global Rating of Overall Well-Being (0–4)	2.5 ± 1.1, 3.0 (2.0, 3.0)	0.06

Total P-CPQ (0–124)	33.4 ± 20.0, 33.0 (17.0, 45.0)	0.03

Oral Symptoms (0–24)	9.9 ± 5.7, 9.0 (6.0, 12.5)	–0.13

Functional Limitations (0–32)	9.2 ± 6.8, 10.0 (3.0, 12.5)	–0.05

Emotional Well-Being (0–28)	7.2 ± 4.9, 7.0 (4.0, 10.0)	0.14

Social Well-Being (0–40)	7.1 ± 6.7, 7.0 (1.5, 9.5)	0.14

**Family Impact Scale**		

Total FIS (0–56)	12.5 ± 7.9, 11.0 (8.0, 18.0)	0.30*

Parental/Family Activity (0–20)	5.2 ± 4.1, 4.0 (2.0, 7.5)	0.26

Parental Emotions (0–16)	4.4 ± 2.9, 4.0 (2.0, 7.5)	0.09

Family Conflict (0–16)	1.9 ± 2.6, 0.0 (0.0, 2.0)	0.25

Financial Burden (0–4)	1.0 ± 1.1, 1.0 (0.0, 2.0)	0.26


* Significant at p < 0.05. N = 64 children with DFT/dft scores and oral health related quality of life data. P-CPQ: Parental-Caregiver Perceptions Questionnaire. FIS: Family Impact scale.

The FIS scores ranged from 0 to 35, with a mean score of 12.5 (SD ± 7.9) (***Table 3***). A statistically significant positive correlation was observed between child DFT/dft scores and total FIS (R = 0.3, p < 0.05). Child DFT/dft scores were observed to have positive but non-significant correlations with the FIS subscale scores.

In both the unadjusted and adjusted regression analyses, we observed no relationship between the variables child gender, child age, and primary caregiver and child DFT/dft scores (***Table 4***). Exploring the relationship between caregiver age and child DFT/dft scores, in both the unadjusted and adjusted models we observed that DFT/dft scores were lower among children with caregivers 31–40 years compared to children with caregivers 18 to 30 years (ß = –3.3 & –3.8, p < 0.05). However, there was no significant difference in DFT/dft scores among children with caregivers greater than 40 years compared to children with caregivers 18 to 30 years. Evaluating the relationship between caregiver DFT scores and child DFT/dft scores, in the unadjusted model we observed child DFT/dft scores were higher among children with caregivers with DFT scores of 7–11 (ß = 2.8) and >11 (ß = 3.7) compared with children with caregivers with DFT scores < 7 (p < 0.05). In the adjusted model, the association remained statistically significant only among children with caregivers with DFT scores of 7–11 (ß = 2.6, p < 0.01). Assessing the relationship between the OHRQoL scores and child DFT/dft scores, in both the unadjusted and adjusted analyses, a one-point increase in FIS total score was associated with an increase in child DFT/dft score (unadjusted: ß = 0.18, p = 0.04; adjusted: ß = 0.31, p < 0.01). There was no statistically significant association between the P-CPQ total score and child DFT/dft scores.

**Table 4:** Regression analysis of child’s DFT/dft.


	MEAN (SD) OR CORRELATION*	UNADJUSTED MODEL†		ADJUSTED MODEL‡	

		ESTIMATE(95% CI)	*P* VALUE	ESTIMATE(95% CI)	*P* VALUE

**Children**					

Gender					

Male	6.8 (4.7)	Reference		Reference	

Female	6.9 (4.4)	0.2 (–1.9, 2.3)	0.86	0.1 (–2.0, 2.2)	0.94

Age					

0 to 5 years	7.4 (4.4)	Reference		Reference	

6 to 11 years	6.9 (4.8)	–0.4 (–3.6, 2.8)	0.80	–0.4 (–3.3, 2.5)	0.80

12 to 18 years	6.4 (4.3)	–0.9 (–4.1, 2.2)	0.55	0.2 (–2.8, 3.1)	0.91

**Caregivers**					

Age					

18 to 30 years	8.5 (4.0)	Reference		Reference	

31 to 40 years	5.2 (4.0)	–3.3 (–6.3, –0.3)	0.03*	–3.8 (–6.5, –1.1)	<0.01*

>40 years	7.6 (5.2)	–0.9 (–4.2, 2.4)	0.60	0.2 (–2.6, 3.1)	0.87

DFT					

0 to 6 teeth	5.1 (4.4)	Reference		Reference	

7 to 11 teeth	7.9 (3.6)	2.8 (0.4, 5.1)	0.02*	2.6 (0.7, 4.4)	< 0.01*

>11 teeth	8.8 (5.1)	3.7 (0.3, 7.1)	0.03*	2.8 (-0.2, 5.8)	0.07

Surveys completed by					

Mother	6.6 (6.3)	Reference		Reference	

Other caregiver	7.1 (4.2)	0.4 (–4.1, 4.9)	0.85	–2.0 (–5.3, 1.4)	0.26

**OHRQoL**					

Total P-CPQ	R = 0.03	0.01 (–0.06, 0.07)	0.86	–0.06 (–0.13, 0.00)	0.06

Total FIS	R = 0.30	0.18 (0.01, 0.35)	0.04*	0.31 (0.10, 0.53)	<0.01*


^†^ N = 66 for child gender, age; N = 64 for caregiver age and gender, P-CPQ and FIS; N = 58 for caregiver DFT ^‡^ N = 58. * Significant at p < 0.05. DFT/dft: Decayed filled teeth. P-CPQ: Parental-Caregiver Perceptions Questionnaire. FIS: Family Impact scale.

## Discussion

The results of this study reveal high levels of untreated dental decay for the children and their caregivers and an association between child dental health status and family quality of life. Ninety-seven percent of the children in this slum community were observed to have untreated dental decay, and only six children (9%) had filled teeth. Due to the fact that dentition status was recorded for a relatively small sample size of children in primary, mixed, and permanent dentitions and that the “m” component of dmft for children can be unreliable, as it is based on the parent’s or child’s recall, DFT/dft scores (dental caries experience) were therefore used to describe the dental health status of the pediatric population. We observed mean child DFT/dft and mean caregiver DFT scores of 6.8 and 8.7, respectively. Collectively, these results indicate a high level of dental disease and also corroborate a significant relationship between child DFT/dft scores and caregiver DFT scores. Our findings are in agreement with existing studies suggesting that caregiver’s oral health status is a strong predictor of the oral health status of their children [24]. We additionally hypothesized that child dental health status would correlate with the quality of life of both the child and the family. Although we did not find a significant association between total P-CPQ scores and child DFT/dft scores, the oral health status of the children was observed to have a negative association with the quality of life of the family, as reflected in the FIS scores.

The prevalence of dental caries experience among the children living in the Community of Claverito is extremely high compared to the average country levels. A national survey conducted by the Ministry of Health of Peru in 2012–2014 reported the prevalence of dental caries in Peru was 59.1% among children age 0–5 years, 85.6% among children age 6–11 years, and 57.6% among children age 12–18 years [25]. A 2019 study reported on a selection of studies on early childhood caries in Peru published between 2010 and 2019 and found an average dmft between 3.6 and 5 [26]. The prevalence of caries and level of disease among the children in our study was 50%–100% greater than the national averages in Peru.

Only 14 (21%) of the 66 children in our study population exhibited history of extractions or restorations. A recent study evaluating factors that determine access to oral health services among children under twelve years of age in Peru found that wealth index, caregivers’ education level, natural region of residence, and age were significant predisposing factors to access to oral health services [27]. Nationally, roughly 30% of children had access to dental health services, whereas only about 20% of children who lived in jungle regions, who came from the poorest families, or whose caregivers’ highest level of education was primary school or less received dental treatment.

Survey instruments to measure burden of untreated decay on the family are limited. This study provided a unique insight into the impact of child dental health status on the family using the validated FIS survey. As hypothesized, the oral health status of the children was observed to have a negative impact on the quality of life of the family. Our results are similar to those of a Brazilian study which found that severity of dental caries was negatively associated with total FIS scores and with three of the four subscales [28]. Given the high level of dental diseases, further research could assess whether improving access to oral health care of slum communities—like the Community of Claverito—could reduce levels of dental disease and improve family quality of life.

Although we did not observe a significant association between total P-CPQ scores and child dental decay levels, P-CPQ scores among the children of the Community of Claverito appear substantially higher than their domestic peers. A recent survey of 200 children from two public schools and two private schools in Lima, Peru reported an average total P-CPQ score of 15.6, and when stratified by caries experience, children with caries had an average total P-CPQ score of 21.3 [18]. The children of the Community of Claverito had P-CPQ scores 80–150% higher.

Several factors affected the results of this study. Owing to the cross-sectional design, we are only able to report associations and not causal relationships. Additionally, the sample size was limited, which reduced the power of the study. This was due, in part, to the two-day time frame available to complete exams and administer surveys, and it was also due to participant fatigue. Dentistry was among one of eight groups from InterACTION Labs interviewing families in the community, and participants were allowed to consent/assent to individual components of the project, therefore some families declined to participate in the oral health component and a non-response bias may affect our estimates. Furthermore, due to the length of the OHRQoL questionnaires, and in an effort to reduce the burden on the caregivers, a single P-CPQ and FIS survey was completed per household instead of per child. The Generalized Estimating Equations method was utilized to account for the effect of clustering by household. Finally, at the time of the analyses, participant-specific dietary data and fluoride exposure data were not available, and data related to interest in, access to, and utilization of dental services were not captured as part of this study.

Lastly, we recognize that it is conventional to analyze dental health status and OHRQoL data with OHRQoL scores as the outcome variable. Because our study design was cross-sectional, because we were aiming at associations between caries and quality of life, and because our study data set had a multi-level structure with the child as the unit of observation (clustering by household) and quality of life (P-CPQ and FIS) measured at the household level, the child’s DFT/dft score was used as the outcome variable. This analysis approach allowed for the most efficient use of the data by assessing independent associations of P-CPQ and FIS scores with caries, thus including both variables as independent variables in the same model. If the P-CPQ and FIS scores were used as outcome variables, the analysis would have needed to be done at the household level, and this would have resulted in a consequent significant decrease in sample size and a need to summarize the children’s DFT/dft measures at the household level.

In summary, results from this study supported our hypotheses that children and their caregivers living in Community of Claverito have a high level of untreated dental decay, and that the children’s oral health is associated with lower quality of life for their families. There are an estimated 90,000 people living in riverine slum communities in Iquitos that could benefit from an increase access to oral health services. While Claverito is a slum community, and therefore not recognized by the city government, universal healthcare coverage, which includes dental care, is available to all schoolchildren in Peru. Further research to improve availability and access to oral health services are needed to achieve better health for informal communities like the one of Claverito.
